# Quercetin as an Antiviral Agent Inhibits Influenza A Virus (IAV) Entry

**DOI:** 10.3390/v8010006

**Published:** 2015-12-25

**Authors:** Wenjiao Wu, Richan Li, Xianglian Li, Jian He, Shibo Jiang, Shuwen Liu, Jie Yang

**Affiliations:** 1 State Key Laboratory of Organ Failure Research, Guangdong Provincial Key Laboratory of New Drug Screening, School of Pharmaceutical Sciences, Southern Medical University, Guangzhou 510515, China; wj910103@126.com (W.W.); levine091@163.com (R.L.); herelxl@163.com (X.L.); jianhe@smu.edu.cn (J.H.); 2 Lindsley F. Kimball Research Institute, New York Blood Center, New York, NY 10065, USA; shibojiang@fudan.edu.cn; 3 Key Lab of Medical Molecular Virology of Ministries of Education and Health, Shanghai Medical College, Fudan University, Shanghai 200032, China

**Keywords:** entry inhibitor, hemagglutinin, influenza A virus, quercetin

## Abstract

Influenza A viruses (IAVs) cause seasonal pandemics and epidemics with high morbidity and mortality, which calls for effective anti-IAV agents. The glycoprotein hemagglutinin of influenza virus plays a crucial role in the initial stage of virus infection, making it a potential target for anti-influenza therapeutics development. Here we found that quercetin inhibited influenza infection with a wide spectrum of strains, including A/Puerto Rico/8/34 (H1N1), A/FM-1/47/1 (H1N1), and A/Aichi/2/68 (H3N2) with half maximal inhibitory concentration (IC_50_) of 7.756 ± 1.097, 6.225 ± 0.467, and 2.738 ± 1.931 μg/mL, respectively. Mechanism studies identified that quercetin showed interaction with the HA2 subunit. Moreover, quercetin could inhibit the entry of the H5N1 virus using the pseudovirus-based drug screening system. This study indicates that quercetin showing inhibitory activity in the early stage of influenza infection provides a future therapeutic option to develop effective, safe and affordable natural products for the treatment and prophylaxis of IAV infections.

## 1. Introduction

Influenza A viruses (IAVs) cause worldwide outbreaks and seasonal pandemics, seriously impacting public health, as well as the economy. The H1N1 subtype of swine influenza lineages has continued to circulate in humans, and serious concerns have been raised over its pandemic potential [1]. Additionally, the highly pathogenic avian influenza A (H5N1) virus causes acute respiratory distress syndrome and multi-organ failure with approximately 60% lethality [2]. The re-assorted influenza A virus (H7N9) has led to extra pulmonary complications with a fatality rate of more than 34% since the first case of the disease was found in China [3]. Genetic variation of influenza A viruses has resulted from genetic drift and genetic shift caused by selective pressure from the environment or host immune response, making it almost impossible to produce a timely and sufficiently effective vaccine to prevent epidemic outbreaks [4]. Consequently, development of novel strategies is urgently needed to prevent further spread of influenza A virus, among which anti-influenza agent is considered as the most effective intervention tool to control virus infections.

Currently, two classes of anti-influenza drugs are available, one targeting the matrix 2 (M2) ion channel and the other targeting neuraminidase (NA) expressed on the virus envelope. M2 ion-channel inhibitors, such as amantadine (trade name: Symmetrel) and rimantadine (trade name: Flumadine), are only effective against type A virus, but these drugs have been reported to cause neurological side effects and widespread drug resistance [5]. Neuraminidase inhibitors (NAIs), such as oseltamivir (trade name: Tamiflu) and zanamivir (trade name: Relenza), were recommended for treatment and prevention of acute uncomplicated flu caused by influenza A and B [6]. Peramivir (trade name: Rapivab) is the only intravenous formulation among anti-influenza neuraminidase inhibitors currently available [7]. Unfortunately, continually emerging NAI resistance limits their development and effectiveness. Also, dual resistance to both oseltamivir and amantadine has been detected [6,8,9,10,11]. Thus, there is a strong need to explore new antiviral drugs against influenza virus.

Virus entry is the initial step of the viral replication cycle; prevention of viral entry leads to suppression of viral infectivity and is an attractive antiviral strategy [12]. The influenza virus envelope protein hemagglutinin (HA) plays critical roles in viral entry [13]. Mature HA is a homotrimer, and each monomer is composed of two disulfide-linked subunits, HA1 and HA2, generated by proteolytic cleavage of the primary translation product HA0 and modification by multiple glycosylations [13]. HA is responsible for the binding of the virus to host cells and subsequent membrane fusion within the late endosome. Most of the HA1 subunit in the head region of HA is responsible for viral binding to the cell surface. Following binding, the virus is internalized by endocytosis. Within the low-pH (5.0 to 5.5) environment of the endosome, HA undergoes conformational rearrangements, resulting in exposure of the fusion peptide, which enters the endosomal membrane of the host cell, and the HA2 subunit in the stem region leads to viral-cell membrane fusion. After fusion, the viral ribonucleic proteins (vRNPs) are released into the cytosol and transported into the nucleus, where replication occurs. Under the hydrolyzation by NA, the newborn virions are released from the infected cells.

Natural products appear to be a major source of anti-influenza drug discovery and offer new prospects for influenza management [14,15,16,17,18]. Quercetin (3,3’,4’,5,7-pentahydroxyflavone) is one of the most ubiquitous flavonoids, and it is found in many Chinese herbs, vegetables and fruits, as well as red wine [19]. Numerous experiments have shown that quercetin exerts antiproliferative [20], antioxidative [21], antibacterial [22], anticancer [23] and antiviral [24] effects. Uchide *et al.* [25] found that quercetin could protect patients from dying from severe complications associated with the pandemic influenza A (H1N1) virus infection. However, the details of its anti-influenza mechanism remain to be elucidated. To account for this, we investigate potential inhibition mechanism of quercetin against the influenza virus and hope to develop a novel anti-IAV agent against the influenza virus.

## 2. Result

### 2.1. Quercetin Inhibited Influenza A Virus Infection

**Figure 1:**
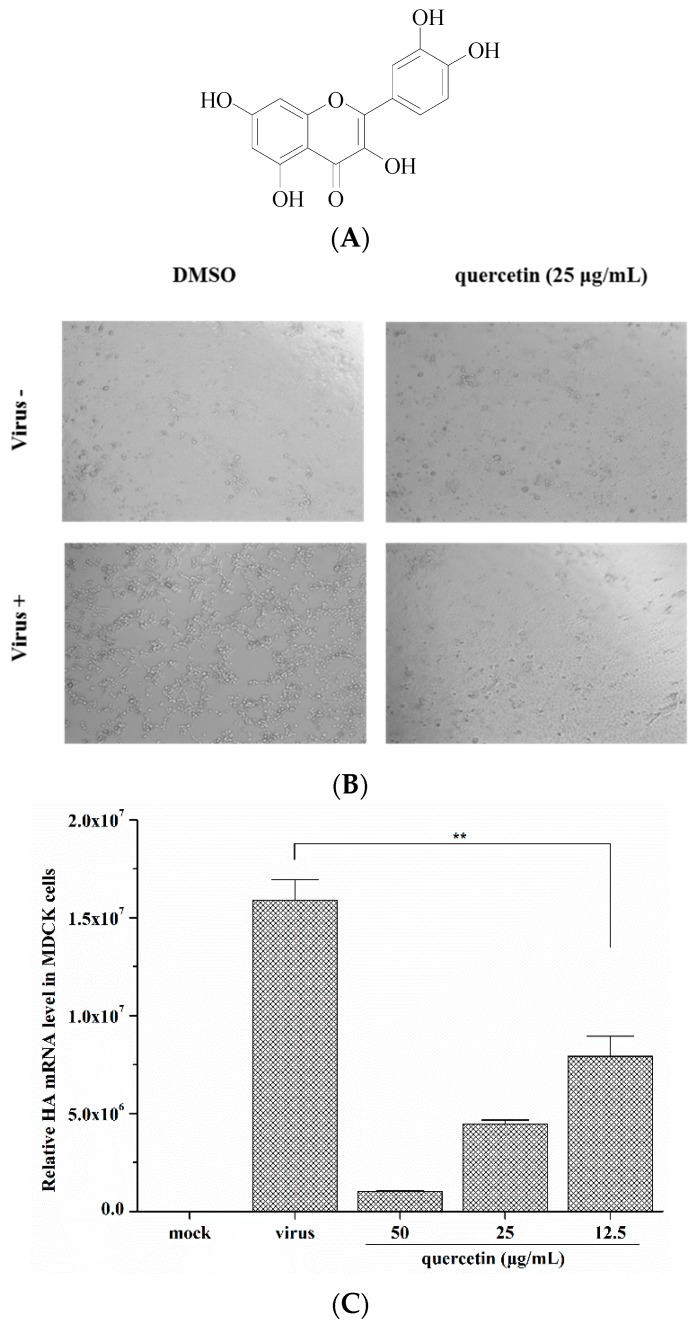

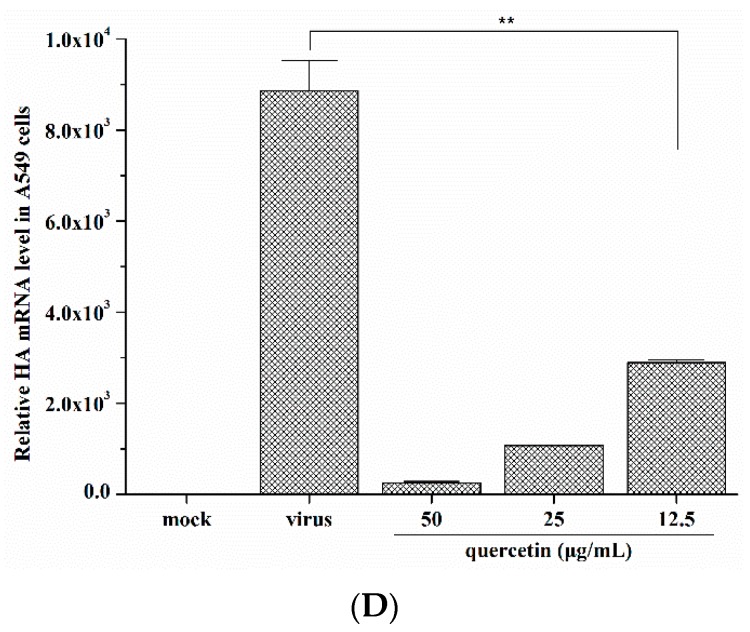
(**A**) The chemical structure of quercetin; (**B**) Validation of the protection of MDCK cells from influenza A/Puerto Rico/8/34 (H1N1) infection by quercetin. Original magnification, 40 ×. (**C**,**D**) The inhibitory effect of quercetin on the influenza A/Puerto Rico/8/34 (H1N1) HA mRNA expression in MDCK cells and A549 cells were detected by quantitative real-time PCR at 24 h post-infection. ** *p* < 0.05.

The anti-influenza viral activity of quercetin against influenza A virus was evaluated by measuring the inhibition on cell infection model. MDCK cells were infected with the influenza virus with the presence of quercetin, and the cytopathic effect (CPE) was observed at 48 h post-infection. In parallel, the inhibition rate was represented by the determination of the cell viability (CPE inhibition, Figure 1B) using MTT assay. The data indicated that quercetin exerted an obvious inhibitory effect for both H1N1 and H3N2 virus strain infections in a dose-dependent manner, and the IC_50_ and IC_90_ values were listed in Table 1. Considering the inhibition rate observed in inhibition against the influenza virus A/Puerto Rico/8/34 (H1N1) was relatively lower than other strains, we subsequently explored the mechanism of its anti-influenza activity with the influenza A/Puerto Rico/8/34 (H1N1) virus strain.

**Table 1:** IC_50_ and IC_90_ of quercetin against influenza A virus strains.

Influenza A Virus Strains	Inhibitory Activity (Mean ± S.D.) ^a^	
	Quercetin	
	IC_50_ (µg/mL)	IC_90_ (µg/mL)
A/Puerto Rico/8/34 (H1N1)	7.76 ± 1.10	24.58 ± 6.71
A/FM-1/47/1 (H1N1)	6.23 ± 0.47	20.47 ± 3.97
A/Aichi/2/68 (H3N2)	2.74 ± 1.93	8.24 ± 2.84

^a^ The samples were tested in triplicate, and the data were presented in mean ± S.D. This experiment was repeated three times with similar results. IC_50_: half maximal inhibitory concentration; IC_90_: ninety percent inhibitory concentration.

To investigate the inhibitory effect of quercetin on influenza virus infections, we conducted a quantitative real-time PCR to determine quercetin’s inhibition on the synthesis of viral mRNA in MDCK cells and A549 cells. Cells were infected with the influenza A/Puerto Rico/8/34 (H1N1) virus at 100 TCID_50_ in the presence of quercetin as described in the experimental section, and the total RNA was isolated from the infected cells and analysed by quantitative real-time PCR using primers specific for the viral HA mRNA at 24 h post-infection. The result revealed that quercetin reduced HA mRNA transcription in influenza-virus-infected cells in a dose-dependent manner (Figure 1C,D).

**Figure 2:**
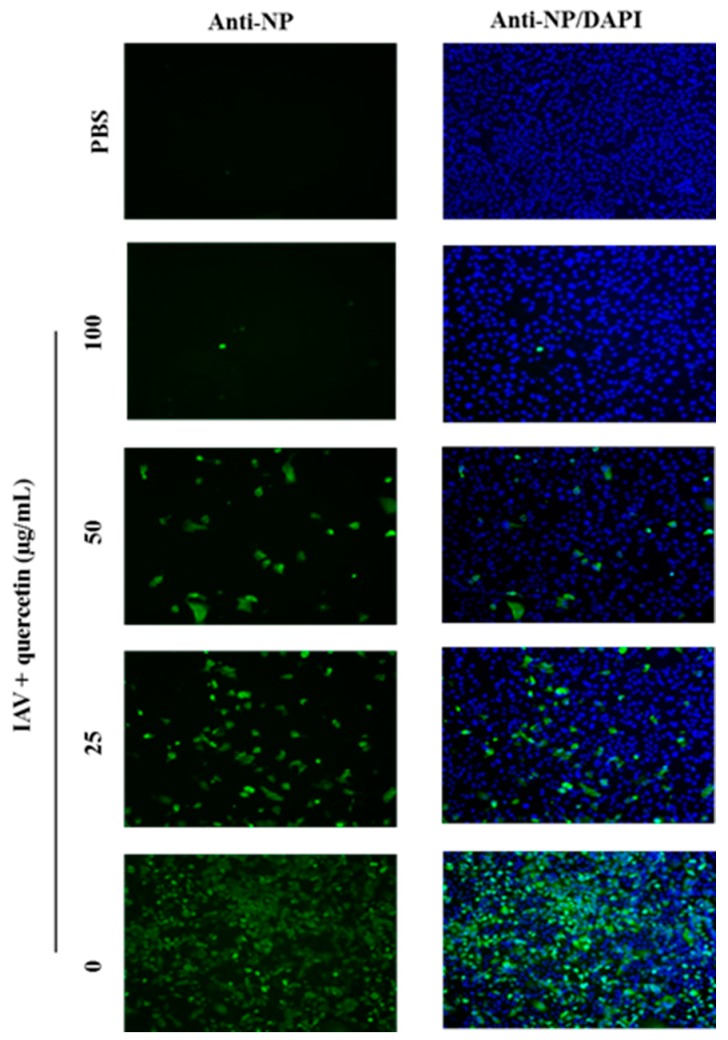
Quercetin inhibited vRNP localization in the nucleus. MDCK cells were infected with virus in the presence of quercetin at the concentration of 100, 50 and 25 µg/mL. Viral NP protein was detected with NP-specific monoclonal antibody and Alexa 488-conjugated goat anti-mouse secondary antibody (**green**); the nuclei were counterstained with DAPI (**blue**). Original magnification, 40×.

To further confirm that quercetin also impairs viral protein expressions, MDCK cells were infected with the influenza A/Puerto Rico/8/34 (H1N1) virus at 100 TCID_50_ in the presence of various concentrations of quercetin. At 24 h post-infection, cells were analyzed for virus nucleoprotein (NP) localization by an indirect immunofluorescence assay. As the data shown (Figure 2), quercetin showed significant inhibition on viral NP protein synthesis in a dose-dependent way in MDCK cells.

All the data above adequately illustrated quercetin could effectively inhibit influenza virus infections and inspired us to further detect the mechanism of quercetin inhibiting influenza virus infections.

### 2.2. Quercetin Performed the Inhibitory Activity in the Initial Stage of Influenza Virus Infection

**Figure 3:**
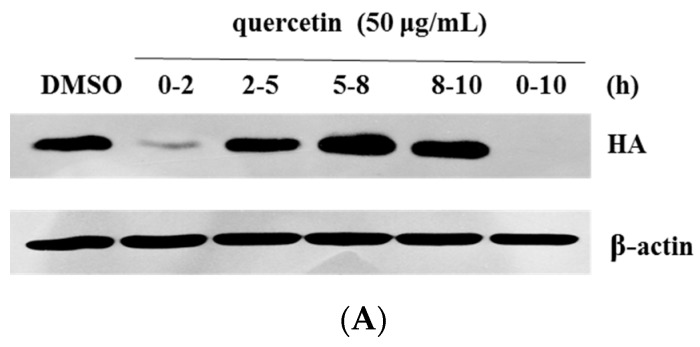

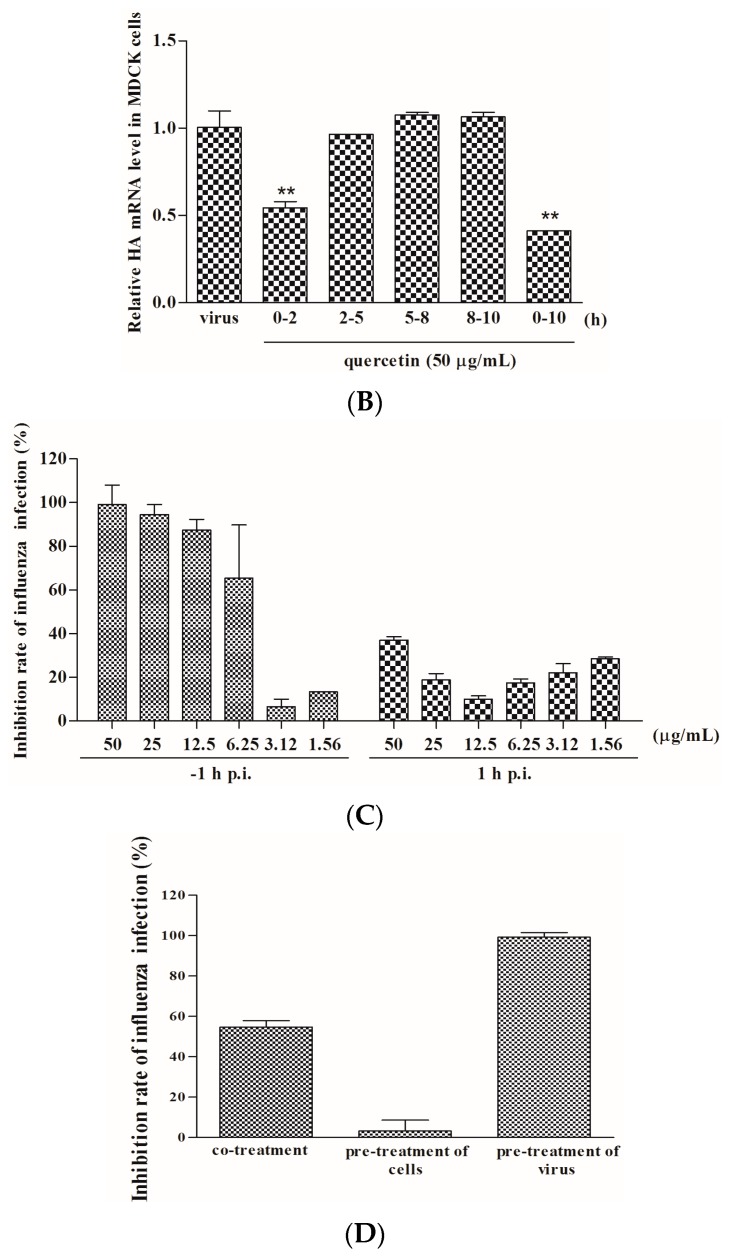
Quercetin performed the inhibitory activity in the initial stage of influenza virus infection. (**A**) The cells were infected with influenza A/Puerto Rico/8/34, and the virus-infected cells were then treated with quercetin in 0–2 h, 2–5 h, 5–8 h, 8–10 h and 0–10 h time intervals respectively. At 10 h post-infection, the viral HA protein was detected by Western blotting; (**B**) The cells were infected with influenza A/Puerto Rico/8/34, and the cells were then treated with quercetin in 0–2 h, 2–5 h, 5–8 h, 8–10 h and 0–10 h time intervals respectively. The viral HA mRNA was detected by quantitative real-time PCR at 10 h post-infection; (**C**) Serially diluted compound was added 1 h before virus infection (−1 h p.i.) or 1 h after virus infection (1 h p.i.). The extent of virus infection in the cell was determined at 48 h post-infection using MTT assay; (**D**) Different modes of treatment, namely co-treatment, pre-treatment of cells and pre-treatment of virus, were conducted to clarify whether the quercetin targets the host cell or the influenza virus. The protection of quercetin to the virus-infected cell was detected at 48 h post-infection by MTT assay.

Having identified the anti-influenza activity of quercetin, we hoped to explore the potential mechanism of its anti-influenza activity. As Yu *et al.* [26] reported, one life cycle of the influenza virus was around 8–10 h, and was divided into three steps: virus entry (0–2 h), viral genome replication and translation (2–8 h), and progeny virion release (8–10 h). To identify which step(s) quercetin target(s) during influenza virus infections, we performed a time-of-addition assay to study the inhibitory effect of quercetin. The infected cells were treated with quercetin in 0–2 h, 2–5 h, 5–8 h, 8–10 h and 0–10 h time intervals during virus infection respectively, then the HA protein and mRNA expression levels were detected by Western blotting and quantitative real-time PCR, which represented the extent of virus infection. As Figure 3A,B shows, quercetin inhibited viral HA expression mainly in the 0–2 h (the entry step) and 0–10 h (the whole life cycle) time intervals, while the inhibitory effect during other time intervals was not clear. This data demonstrated that quercetin may inhibit virus infections in the early stage covering viral attachment, endocytosis and viral-cell fusion. Additionally, we conducted another relevant test to determine the effect of quercetin on virus entry (Figure 3C). A serially diluted compound was added to the cells during the time of virus absorption (−1 h p.i. (post infection)) or at 1 h post-infection (1 h p.i.). For the former, infection inoculum containing the test compound was replaced with a fresh medium containing 1 μg/mL TPCK-trypsin after 1 h incubation; for the latter, the compound was added after virus infection. At 48 h post-infection, the inhibition rates of influenza infection were detected through MTT assay. The results demonstrated that quercetin inhibited virus infection when it was added during the process of virus infection rather than post-virus infection.

In addition, to determine whether quercetin targets the host cell or the virion, we designed three modes of treatment assay as previously reported with some modification [26]. As Figure 3D shows, in the first mode of treatment, namely co-treatment, MDCK cells were inoculated with the influenza virus in the presence of quercetin at 4 °C for 1 h and subsequently washed to remove unbound viruses and compounds. For the second mode, namely pre-treatment of cells, cells were incubated with quercetin at 4 °C for 30 min and subsequently washed to remove the unbound compounds; the pretreated cells were exposed to viruses for 1 h and subsequently washed to remove unbound viruses. In the third experiment, which was called the pre-treatment of the virus, the virus was incubated with quercetin at 4 °C for 30 min, and the unbound compounds were subsequently removed from the virus by ultrafiltration. The host cells were inoculated with the pretreated viruses for 1 h and subsequently washed to remove unbound viruses. All of the cells treated above were then further cultured at 37 °C for 48 h, and the inhibitory effect was then measured by MTT assay. According to the data (Figure 3C), the co-treatment with quercetin, at the concentration of 50 µg/mL, exerted an inhibition rate about 49.21%, and the pre-treatment of the virus led to an inhibition rate of 99.30%, while pre-treatment of the cells had almost no detectable effect. We concluded that quercetin may target the influenza virus particles rather than the host cells.

### 2.3. Quercetin Showed Binding Affinity to Influenza HA Protein

**Figure 4:**
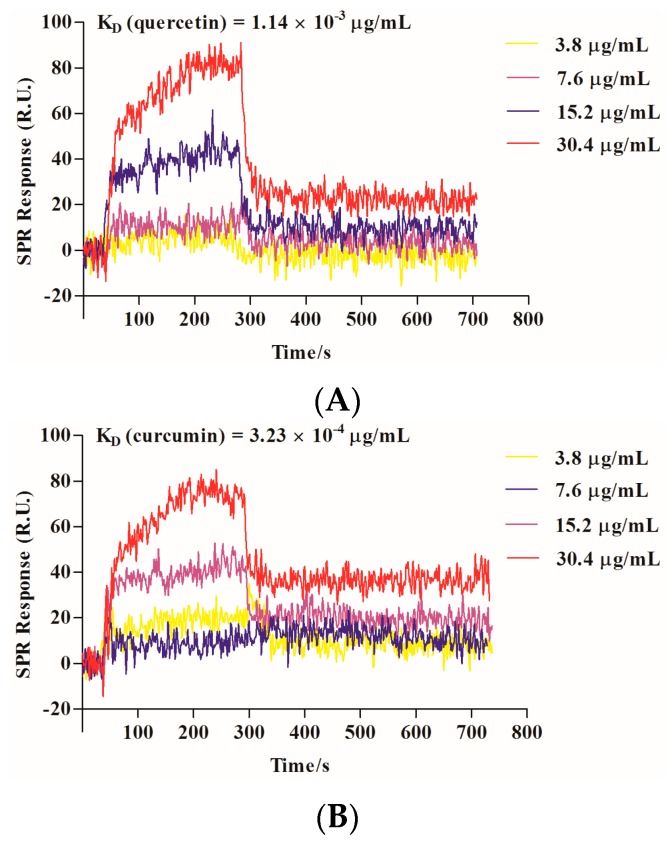
The interaction between the influenza HA and quercetin was analyzed with PlexArray HT systems. (**A**) The binding curve of quercetin with HA protein in SPR assay; (**B**) The binding curve of curcumin with HA protein in SPR assay. The compounds were immobilized on a sensor chip, and the recombinant influenza HA protein was injected as analytes at various concentrations dissolved in a running buffer at a flow rate of 3 μL/s. The contact time and dissociation time were 300 s respectively. The chip platform was regenerated with gly-HCl (pH 2.0) and washed with the running buffer. Curcumin was used as a positive control in this assay. The affinity constant K_D_ is the ratio of dissociation constant K_d_ with association constant K_a_, K_D_ = K_d_/K_a_.

**Figure 5:**
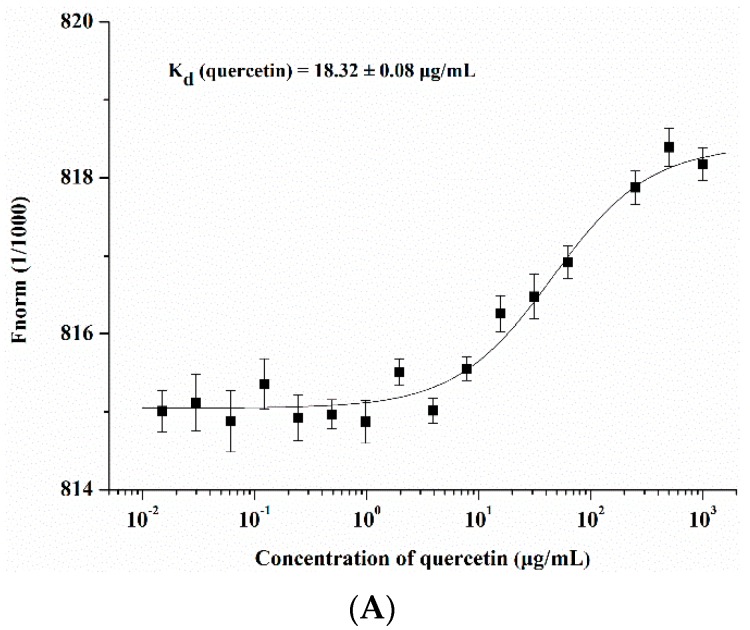

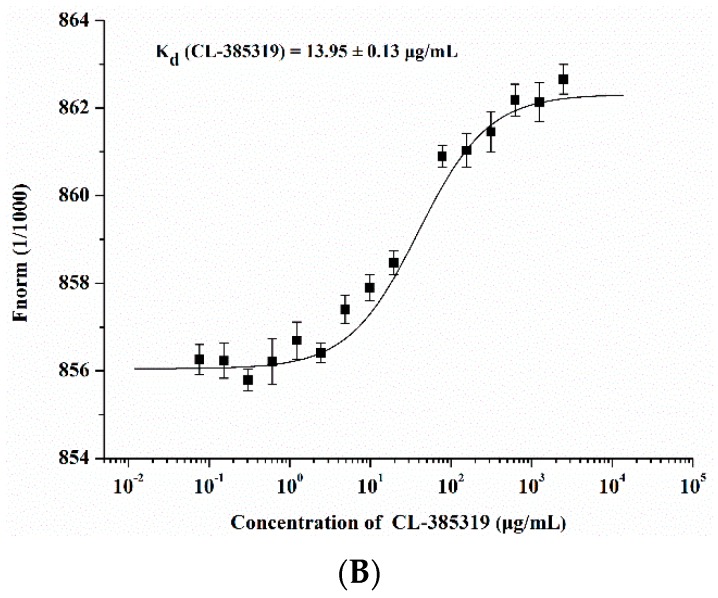
Protein-binding assays in biological liquids using microscale thermophoresis (MST). (**A**) The binding curve of quercetin with NT647 labeled recombinant influenza HA protein; (**B**) The binding curve of CL-385319 with NT647 labeled recombinant influenza HA protein. NT647 (NanoTemper Technologies) labeled recombinant influenza HA protein was mixed with two-fold diluted compounds in the same volume as that of the final concentrations. MST experiments were performed on a Monolith NT.115 system (NanoTemper Technologies) using 100% LED and 20% IR-laser power. The compound CL-385319 was used as the positive control. K_d_ means the dissociation constant.

The viral envelop protein hemagglutinin plays a critical role in virus entry. Mature HA is composed of two disulfide-linked subunits, HA1 and HA2. HA1 is responsible for virus binding, and the HA2 subunit is responsible for viral-cell fusion. We speculated that there was a binding affinity between HA protein and quercetin and confirmed it through a surface plasmon resonance (SPR) assay, which was designed to detect the binding between two molecules. Quercetin showed medium-binding affinity to HA protein with a K_D_ value of 1.14 × 10^−3^ µg/mL. Cucurmin, which was shown to possess anti-influenza virus entry activity [27], was used as a positive control with a K_D_ value of 3.23 × 10^−4^ µg/mL in our assay (Figure 4). To further confirm the binding affinity between quercetin and HA protein, we conducted a microscale thermophoresis (MST) assay that allowed for a quantitative analysis of interaction between small molecules and protein in-free solutions, and with low sample consumption. The interaction between small molecules and protein induces molecular changes in size, charge, hydration shell and conformation, which will affect the molecule mobility during the process of thermophoresis. The results demonstrated that quercetin interacted with influenza HA protein in a dose-dependent manner. The compound CL-385319, which was reported previously to have interacted with the influenza HA protein [28], was used as a positive control (Figure 5).

### 2.4. Quercetin Inhibited Influenza Virus HA Mediated Hemolysis

**Figure 6:**
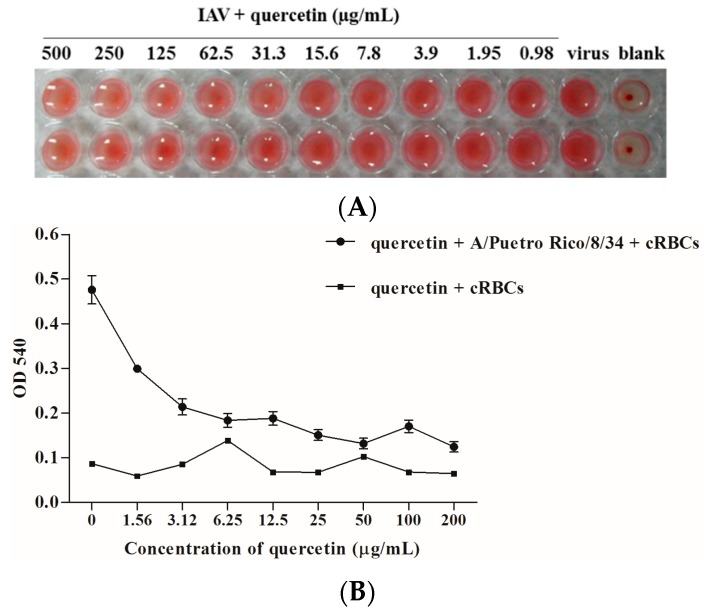
(**A**) Hemagglutinin inhibition assay for quercetin. 4HAU influenza A/Puerto Rico/8/34 (H1N1) virus was incubated with serally diluted quercetin at room temperature for 30 min. 0.5% cRBCs was added, and the virus then incubated at room temperature for 1 h. Note the inhibition on hemagglutination of quercetin; (**B**) Hemolysis inhibition assay of quercetin against the influenza A/Puerto Rico/8/34 (H1N1) virus strain. Quercetin diluted in PBS was mixed with an equal volume of the influenza virus A/Puerto Rico/8/34 (H1N1) in a 96-deep well plate. After incubating at room temperature for 30 min, 2% chicken erythrocytes pre-warmed at 37 °C were added. The mixture was incubated at 37 °C for another 30 min. To trigger hemolysis, sodium acetate (0.5 M; pH 5.0) was added and incubated at 37 °C for 15 min. Plate was centrifuged at the end of incubation at 3000 rpm for 5 min, and the supernatants were transferred to another flat-bottom 96-well plate. The OD_540_ was read on a microtiter plate reader.

In order to detect which domain quercetin targets, we first designed a hemagglutinin inhibition (HI) assay to detect whether quercetin could interfere with the interaction between HA and cellular receptors and subsequently prevent virus attachment to the cell. As the data shows, it had no inhibitory effect on viral attachment to the cell, which was mainly mediated by the HA subunit. We wondered if quercetin could interfere with viral-cell membrane fusion, which was mediated by HA2 subunit. Subsequently, the hemolysis assay was performed with the influenza virus A/Puerto Rico/8/34 (H1N1) to determine the effect of quercetin on fusion. To trigger hemolysis, the virus-cell suspension was briefly acidified (pH 5.0) to initiate HA conformational changes that lyse chicken red blood cells (cRBCs). Wells absent in the virus were used as controls to determine the effect of compounds on cRBCs. The results indicated that quercetin could inhibit influenza-virus-HA-mediated hemolysis in a dose-dependent manner, indicating that quercetin may target the membrane fusion process during virus entry (Figure 6).

### 2.5. Quercetin Inhibited Entry of H5N1 Virus with Divergent Strains

**Figure 7:**
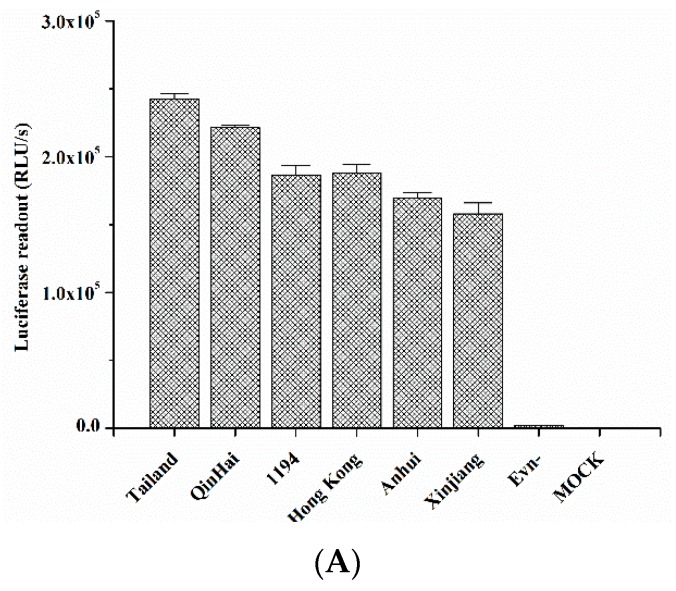

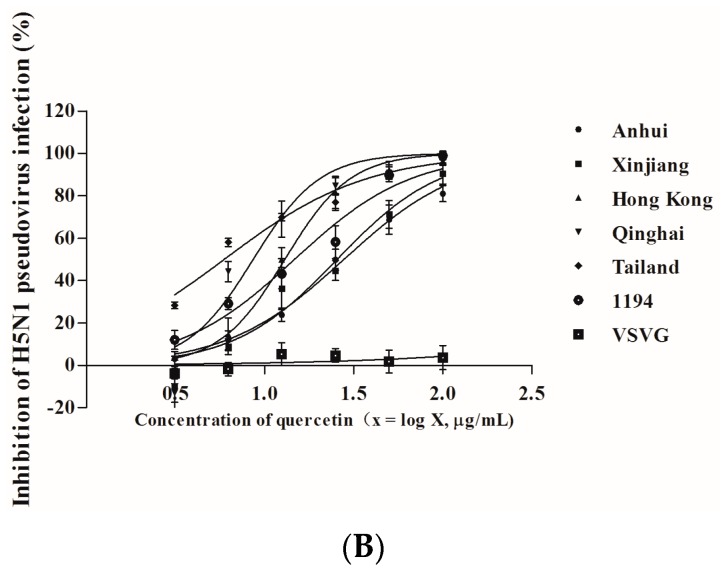
The inhibitory activity of quercetin on H5N1 pseudovirus infection. (**A**) Infectivities of a series of H5N1 pseudoviruses, named A/Anhui/1/2005A, A/Xinjiang/1/2006, A/HongKong/156/1997, A/Qinghai/59/2005, A/Thailand/Kan353/2004, and A/VietNam/1194/2004—Env-pseudovirus and cells-only (mock) were used as the negative control; (**B**) The inhibitory activity of quercetin against the infection of different H5N1 subtype pseudoviruses and VSV-G pseudovirus on MDCK cells.

Using the viral-like particles that pseudotyped with the HIV backbone plasmid and influenza envelop glycoproein HA plasmids from IAV H5N1 virus strains, including A/Anhui/1/2005A, A/Xinjiang/1/2006, A/Hong Kong/156/1997, A/Qinghai/59/2005, A/Thailand/Kan353/2004, and A/VietNam/1194/2004, the results confirmed that quercetin could effectively inhibit the entry of divergent strains of the H5N1 virus (Figure 7). The IC_50_ and IC_90_ values are shown in Table 2. On the contrary, quercetin had no effect on the infection of the VSV-G pseudovirus (Figure 7), demonstrating that quercetin specifically interfered with the function of the H5N1 influenza HA envelope protein. The data reconfirmed the result and confirmed that quercetin could effectively inhibit influenza virus entry through its interaction between HA proteins.

**Table 2:** Inhibitory activities of quercetin on infection by different H5N1 pseudovirus

H5N1 Pseudovirus	Inhibitory Activity (Mean ± S.D.) ^a^	
	Quercetin	
	IC_50_ (µg/mL)	IC_90_ (µg/mL)
A/Anhui/1/2005A	31.47 ± 4.19	127.39 ± 16.37
A/Xinjiang/1/2006	26.67 ± 4.49	92.27 ± 3.29
A/Hong Kong/156/1997	18.90 ± 2.32	53.95 ± 1.84
A/Qinghai/59/2005	12.27 ± 1.30	49.54 ± 4.51
A/Thailand/Kan353/2004	11.89 ± 3.31	31.19 ± 4.82
A/VietNam/1194/2004	17.35 ± 1.58	57.34 ± 4.71

^a^ The samples were tested in triplicate, and the data were presented in mean ± S.D. This experiment was repeated three times with similar results.

The *in vitro* cytotoxicity of quercetin was tested by the MTT assay, and quercetin exhibited no significant cytotoxicity on MDCK cells or A549 cells at the concentrations as high as 250 µg/mL.

## 3. Discussion

Influenza A viruses continue to emerge unpredictably annually and are associated with significant morbidity and mortality, particularly in the circulation of high pathogenic avian influenza H5N1. Unfortunately, side effects and the appearance of drug-resistance of currently available anti-IAV drugs limit their clinical use. Novel anti-influenza virus agents, especially those targeting on newly developed targets remain to be clinically developed.

The life cycle of the influenza virus includes viral attachment, entry, replication, and release. Blockade of viral entry/fusion would certainly be a promising therapeutic strategy in the fight against virus infections, including HIV-1, Middle East respiratory symptom coronavirus (MERS-CoV) and the influenza virus [12,29,30,31]. Remarkably, applications of HIV fusion inhibitors, T20 (enfuvirtide), either alone or in combination with other antivirus agents, have become an attractive antiviral strategy [32]. Moreover, natural small molecules as potential entry inhibitors have been the object of research for many years [28,33,34,35]. However, to date, no drug-blocking viral entry is available for the treatment and prevention of influenza.

In the initial study, we found that quercetin displayed antiviral activity against different influenza virus strains, including H1N1and H3N2, which impelled us to investigate the mechanism of its anti-influenza activity. Surprisingly, we found that the inhibitory effect of quercetin was enhanced when the virus was pre-incubated with quercetin, or the cell was infected with the virus in the presence of quercetin. The time of the additional assay indicated that quercetin effectively inhibited virus infection when it was added during virus entry stage, while the inhibitory effects of other stages were less clear. On the basis of the above results, we wondered whether quercetin targeted the virion or the cell. Through three different modes of treatment, namely co-treatment, pre-treatment of cells and pre-treatment of virus, we found that quercetin targeted influenza viral particles instead of the host cell.

Generally, HA is an essential major glycoprotein on the surface of the influenza virus responsible for entry and fusion of virions [36]. Several studies have identified inhibitors of the receptor binding or membrane fusion activities of HA, particularly the latter [37,38]. Speculating that quercetin could interact with HA and subsequently interfere with virus entry, we used two procedures to assess whether there was a binding affinity between quercetin and HA protein. In the first, the binding affinity was measured by SPR assay, and the result indicated that quercetin showed a medium binding affinity to influenza HA protein. While SPR is an assay that performed by immobilizing one of the binding partners to a thin noble metal film [39], surface immobilization therein may affect the bound molecules’ dynamics and thus alter the binding event. Subsequently, a solution-based MST assay was performed to further confirm the binding affinity between quercetin and HA. As a solution-based method, microscale thermophoresis avoids such surface artifacts and immobilization procedures. Instead, one of the binding partners was fluorescently labeled [40]. The MST assay provided strong support for the result of SPR, confirming that quercetin exhibited a binding affinity to HA. The following design utilized multiple assays to further identify the potential target of quercetin. The HI assay and hemolysis inhibition assay indicated that quercetin efficiently inhibited entry of the influenza A virus, which may be mainly ascribed to its interaction with the HA2 subunit, which mediates the low pH-induced fusion of the viral envelope with the endosomal membrane. Additionally, the influenza H5N1 pseudovirus neutralization assay showed quercetin potently neutralized different strains of HA pseudotyped H5N1 viruses, which reconfirmed that quercetin may target viral surface HA protein and thus inhibit virus infection.

The study indicated that quercetin may exert its antiviral activity via interaction with viral HA protein and then inhibit virus entry into the cell. As we know, an entry inhibitor, named TBHQ, can restrain virus infection at the concentration <20 µM by preventing the conformational change of the HA at a low pH [37]. Whether quercetin could bind to a hydrophobic pocket formed at an interface between HA monomers like TBHQ [37] or other mechanisms to inhibit influenza virus entry, further studies to unravel the structure–activity relationship are underway in our laboratory.

On the other hand, combination applications of multiple therapeutic methods provide several advantages over the single-agent treatment, such as enhanced antiviral potency, reduced drug dosage, delayed emergence of drug resistance, and fewer side effect [41,42]. Since the antiviral mechanism of quercetin was different from the commonly prescribed anti-influenza drug, it may prove to be effective when used in combination with other anti-retroviral agents. We will explore the potential synergistic antiviral effect of quercetin and other antiretroviral agents *in vitro* and *in vivo*. Furthermore, it is noteworthy that quercetin is also available as a dietary supplement with daily doses of 200–1200 mg (manufacturers’ recommendations) in some countries, and it may present its potential as animal feed additives to reduce the risk of avian influenza virus infection in poultry farms. Accordingly, quercetin will be a new addition to the anti-influenza treatments if continuous efforts are made in the modification of quercetin to enhance its pharmacokinetics and pharmacodynamics.

Our study demonstrates quercetin is a novel antiviral agent, which may be used as an effective, safe and affordable chemoprophylaxis or treatment of influenza infection. Importantly, quercetin targeting to the HA2 subunit of influenza hemagglutinin provides new insights for the development of a class of anti-influenza fusion inhibitors.

## 4. Materials and Methods

### 4.1. Materials

293T cells, Madin Darby Canine Kidney (MDCK) cells and human lung epithelial A549 cells were obtained from the American Type Culture Collection (ATCC, Manassas, VA, USA). 293T cells and MDCK cells were grown in Dulbecco’s modified Eagle’s medium (DMEM) supplemented with 10% fetal bovine serum (FBS) and 1% penicillin/streptomycin. A549 cells were maintained in RPMI1640 medium containing 10% FBS and 1% penicillin/streptomycin. Virus stocks of these laboratory-adapted strains including A/Puerto Rico/8/34 (H1N1), A/FM-1/47/1 (H1N1), and A/Aichi/2/68 (H3N2) were propagated in the allantoic cavities of 9-day-old embryonated hen eggs at 37 °C for two days. Afterward, allantoic fluid was harvested, centrifuged at 2000 rpm for 10 min and stored at −80 °C until use. The virus titer was determined through the analysis of the 50% tissue culture infective dose (TCID_50_) and evaluated using the method developed by Reed and Muench [43]. Quercetin was purchased from Must Bio-Tech (Chengdu, China) with a purity of 99.35%, CL-385319 was synthetized in our laboratory with a purity of more than 98%, and curcumin was bought from Sigma-Aldrich (St. Louis, MO, USA).

### 4.2. Cytopathic Effect (CPE) Reduction Assay

Antiviral activity of quercetin was then evaluated by the cytopathic effect reduction assay as previously reported [26] with some modifications. Briefly, MDCK cells were seeded into a 96-well plate at 2 × 10^4^ cells/well and incubated overnight until grown to 90% confluence. Serially diluted quercetin was pre-incubated with the virus at 100 TCID_50_ at 37 °C for 30 min. The cells were washed twice with phosphate buffered saline (PBS) and inoculated with virus-compound mixture at 37 °C for another 30 min. After inoculation, the cells were washed to remove the unabsorbed virus and sustained in serum-free DMEM supplemented with 1 μg/mL l-(tosylamido-2-phenyl) ethyl chloromethyl ketone (TPCK)—trypsin. At 48 h post-infection, the inhibition of viral replication was evaluated by determining the protection against the virus-induced cytopathic effect through microscopy, and the IC_50_ values were calculated by the formazan-based 3-(4,5-dimethylthiazol-2-yl)-2,5-diphenyltetrazolium bromide (MTT) cell viability assessing assay. The experiment was repeated at least three times.

### 4.3. Quantitative Real-Time PCR

To confirm the inhibitory activity, viral HA gene replication in MDCK cells was detected by quantitative real-time PCR [44,45]. Briefly, confluent cell monolayer in a 6-well plate was infected with the influenza A/Puerto Rico/8/34 (H1N1) virus, which was pretreated with quercetin as described before. After infection, the cells were washed twice with PBS to remove unabsorbed virus and sustained in serum-free DMEM supplemented with 1 μg/mL TPCK-trypsin. At 24 h post-infection, the total mRNA was extracted with TRIzol reagent and subsequently reverse-transcribed into cDNA using PrimeScript RT reagent kit (TaKaRa, Dalian, China) according to the manufacturer’s instructions.

The quantitative real-time PCR was completed in an ABI7500 real-time PCR instrument (Applied Biosystems, Foster, CA, USA) with SYBR Premix Ex Taq (TaKaRa). Reference gene antiglyceraldehyde 3-phosphate dehydrogenase (GAPDH) was used for normalization of the HA gene mRNA expression levels. Fold changes of HA gene expression were calculated using a classical 2^−ΔΔ*C*T^ method. Similarly, the experiment was conducted on the A549 cell line to indicate that there was no cell line selectivity for the quercetin’s antiviral activity. All samples were run in triplicate. The primer sequences for each gene are listed in Table 3.

**Table 3:** Primer sequences for quantitative real time PCR.

Target Gene	Sequence
HA-Forward	5′-TTCCCAAGATCCATCCGGCAA-3′
HA-Reverse	5′-CCTGCTCGAAGACAGCCACAACG-3′
GAPDH-Forward	5′-AGGGCAATGCCAGCCCCAGCG-3′
GAPDH-Reverse	5′-AGGCGTCGGAGGGCCCCCTC-3′

### 4.4. Indirect Immunofluorescence Microscopy

Confluent MDCK cells in a 48-well plate were infected with the quercetin-pretreated influenza A/Puerto Rico/8/34 (H1N1) virus as described before. At 24 h post-infection, the supernatant was aspirated, and the cells were washed with PBS. Next, the cells were fixed with 4% paraformaldehyde at room temperature for 10 min and blocked with 3% albumin from bovine serum (BSA) at 37 °C for 1 h. After incubation with nucleoprotein (NP) antibody (1:250 diluted in 3% BSA; Santa Cruz, CA, USA) at 4 °C overnight, cells were incubated with fluorescein isothiocyanat (FITC)-labeled secondary antibody (1:250 diluted in 3% BSA for 1 h at room temperature). The nucleus was stained with 4’,6-diamidino-2-phenylindole (DAPI) at room temperature for 10 min [45]. Fluorescence was observed using an inverted fluorescence microscope (Nikon Eclipse Ti-SAM, Tokyo, Japan). In total, three independent experiments were performed, during which at least three photographs were randomly taken for each well.

### 4.5. Time-of-Addition Assay

To investigate which stage(s) quercetin had an effect on, we conducted a time-of-addition assay as Yu *et al.* [26] reported. The monolayer of MDCK cells grown in a 6-well plate were infected with the influenza A/Puerto Rico/8/34 (H1N1) virus and treated with quercetin at the concentration of 50 µg/mL in different time intervals, turn for 0–2 h, 2–5 h, 5–8 h, 8–10 h, and 0–10 h. At 10 h post-infection, the total protein and mRNA were isolated from cells by radio immune precipitation assay (RIPA) lysis buffer containing protease and phosphatase inhibitors [46] as well as TRIzol reagent as described before. Equal amounts of protein were resolved by 10% SDS-PAGE and transferred to polyvinylidene difluoride (PVDF) membranes (Roche, Indianapolis, IN, USA). Mouse monoclonal anti-HA (1:100 dilution; Sino Biological, Beijing, China) and rabbit monoclonal anti β-actin antibody (1:1000 dilution) were used as the primary antibodies. Anti-mouse or anti-rabbit alkaline phosphatase (AP)-conjugated antibodies were used as the secondary antibodies (1:2000 dilution). Chemiluminescent signals were then developed with Lumiglo reagent (Cell Signaling Technology, Danvers, MA, USA) and exposed to X-ray film (Fujifilm Europe GmbH, Dusseldorf, Germany). At the same time, the HA gene mRNA level was detected via quantitative real-time PCR, which was completed in an ABI7500 real-time PCR instrument (Applied Biosystems, Foster, CA, USA) as described above.

### 4.6. Surface Plasmon Resonance (SPR) Analysis

Interactions between the influenza HA and quercetin were analyzed by the PlexArray^®^ HT system (Plexera® Bioscience, Beijing, China) at room temperature. Quercetin was immobilized on a sensor chip, and, subsequently, recombinant influenza HA (Sino Biological Inc.; Beijing, China) was injected as analytes at various concentrations, using phosphate buffered saline with Tween-20 (PBS-T) (10 mM phosphate buffer with 2.7 mM KCl and 137 mM NaCl, 0.05% Tween 20, pH 4.5) as a running buffer. For binding studies, protein was applied at corresponding concentrations in the running buffer at a flow rate of 3 μL/s with a contact time of 300 s and a dissociation time of 300 s. Chip platform was regenerated with gly-HCl (pH 2.0) and washed with the running buffer. All possible binding curves were fitted by Langmuir equation, and the K_D_ was figured out.

### 4.7. Microscale Thermophoresis (MST) Protein Binding Study

Influenza HA protein was labeled with NT647 (NanoTemper Technologies, München, Bavaria, Germany) and applied at a final concentration of 40 nM. The corresponding unlabeled binding partner (quercetin) was titrated in a two-fold dilution series in a PBS-T buffer containing 5% dimethyl sulfoxide (DMSO) (pH 4.5). Subsequently, 10 µL of each dilution point was mixed with 10 µL labeled HA protein solutions and incubated at room temperature for 10 min. Samples were filled into hydrophilic capillaries (NanoTemper Technologies) for measurement. MST experiments were performed on a Monolith NT.115 system (NanoTemper Technologies) with a 100% light-emitting diode (LED) and 20% infrared radiation (IR)-laser power. Laser on and off times were set at 30 s and 5 s, respectively.

### 4.8. Hemagglutination Inhibition (HI) Assay

The HI assay was conducted to evaluate the inhibitory effect of quercetin on HA1 subunit mediated viral adsorption to the cellular sialic acid receptor. The lowest viral titer causing the aggregation of chicken red blood cells (cRBCs) was defined as 1 HA Unit (HAU), and a virus titer of 4 HAUs was used for the assay. In the HI assay, two-fold serially diluted quercetin (25 µL) was mixed with the same volume as that of the influenza virus A/Puerto Rico/8/34 (H1N1) at room temperature for 30 min. This mixture was subsequently incubated with 50 µL of 0.5% cRBCs at room temperature and the inhibition hemagglutination of quercetin was observed at 4 °C for 1 h. The virus in the absence of quercetin was used as the negative control, and PBS was used as the blank control.

### 4.9. Hemolysis Inhibition Assay

In accordance with Luo *et al.* [47], cRBCs were washed with PBS and stored in Alsever’s solution at 4 °C until use [48]. 100 µL of a compound diluted in PBS was mixed with the influenza virus A/Puerto Rico/8/34 (H1N1) strain (10^7^ TCID_50_/mL) of equal volume. After incubation of the virus-compound mixture at room temperature for 30 min, 200 µL of pre-warmed 2% cRBCs were added to the mixture. The mixture was incubated at 37 °C for another 30 min. Afterward, 100 µL of sodium acetate (0.5 M; pH 5.0) was added and mixed well with the erythrocyte suspension to trigger hemolysis, and subsequently allowed to incubate at 37 °C for 15 min. Plates were centrifuged at the end of the incubation at 3000 rpm for 5 min to separate nonlysed erythrocytes. 200 µL of supernatant was transferred to another flat-bottom 96-well plate. The optical density at 540 nm (OD_540_) was detected on a micro titer plate reader.

### 4.10. HA Pseudovirus Neutralization Assay

Avian influenza pseudoviruses expressing H5 HA (HIV (human immunodeficiency virus)/HA (H5)) were produced as described previously [49]. Briefly, 293T cells were co-transfected with 10 μg plasmid encoding Env-defective, luciferase-expressing HIV-1 (pNL4-3.luc.RE) and 10 μg each of the 6 plasmids, including QH-HA-, XJ-HA-, AH-HA-, HK97-HA-, 1194-HA- and Tailand-HA-pcDNA3.1, respectively, into 100 mm culture dishes using Polyetherimide (PEI). Exogenous bacterial neuraminidase (NA) from *Vibrio cholerae* (Sigma, St. Louis, MO, USA) was added 24 and 48 h later, and supernatants were harvested 72 h post-transfection for single-cycle infection.

A pseudotyped virus bearing VSV envelope glycoprotein (HIV/VSV-G) was produced in the same way. The pseudovirus titers were quantitated by using luciferase substrate. For the drug screening process, pseudoviruses were incubated with serially diluted compounds at 37 °C for 15 min. The compound-virus mixture was subsequently added to the cells. 48 h later, cells were lysed by cell lysis buffer (Promega, Madison, WI, USA) and transferred to 96-well luminometer plates. Luciferase substrate (Promega) was added, and relative luciferase activity was determined by Ultra 384 luminometer (GENios Pro, TECAN, Bedford, MA, USA).

### 4.11. Cytotoxicity Test

The cytotoxicity of quercetin on MDCK cells and A549 cells was measured by the MTT assay [50]. Briefly, cells (1 × 10^3^/well) were seeded in a 96-well plate and grown overnight. The medium was then replaced with fresh medium containing serially diluted compounds and further incubated at 37 °C with 5% CO_2_ for 48 h. The culture medium was removed, and 100 µL of a 0.5 mg/mL 3-(4,5-dimethylthiozol-2-yl)-3,5-dipheryl tetrazolium bromide (MTT, Sigma-Aldrich) solution was added, followed by incubation at 37 °C for 4 h. Afterward, the supernatants were aspirated, and 150 µL of dimethyl sulfoxide (DMSO) was added to each well. The stained formazan product was determined spectrophotometrically at 570 nm in a microplate reader (GENios Pro, TECAN, Bedford, MA, USA).

### 4.12. Statistical Analysis

The results are shown as mean ± standard deviation (S.D). ANOVA was used to test statistical significance between groups. In all cases, *p* < 0.05 was regarded as statistically significant and *p* < 0.05 was marked with **.

## 5. Conclusions

In our study, we found quercetin possessed anti-influenza activity. The subsequent mechanism study indicated quercetin showed inhibitory effect during virus entry. Then we found quercetin interacted with influenza hemagglutinin protein and then inhibited viral-cell fusion. The study showed quercetin may be developed as a future therapeutic option for the therapy and prophylaxis of IAV infection.
